# Not All SAVR Are Created Equal: All the Approaches Available for Surgical Aortic Valve Replacement

**DOI:** 10.3390/jcdd12030084

**Published:** 2025-02-24

**Authors:** Francesco Cabrucci, Serge Sicouri, Massimo Baudo, Dimitrios E. Magouliotis, Yoshiyuki Yamashita, Beatrice Bacchi, Dario Petrone, Beman Wasef, Aleksander Dokollari, Massimo Bonacchi, Basel Ramlawi

**Affiliations:** 1 Department of Cardiac Surgery Research, Lankenau Institute for Medical Research, Main Line Health, Wynnewood, PA 19096, USA; sicouris@mlhs.org (S.S.); baudom@mlhs.org (M.B.); magouliotisd@mlhs.org (D.E.M.); yamashitay@mlhs.org (Y.Y.); wasefb@mlhs.org (B.W.); ramlawib@mlhs.org (B.R.); 2 Department of Cardiac Surgery, Lankenau Heart Institute, Main Line Health, Wynnewood, PA 19096, USA; 3 F.U. Cardiac Surgery Department, AOU Careggi University Hospital, 50127 Firenze, Italy; beatrice.bacchi@unifi.it (B.B.); dario.petrone@unifi.it (D.P.); massimo.bonacchi@unifi.it (M.B.); 4 Department of Cardiac Surgery, St. Boniface Hospital, University of Manitoba, Winnipeg, MB R2H 2A6, Canada; dokollaria@mlhs.org

**Keywords:** SAVR, mini-sternotomy, right anterior thoracotomy, transcervical SAVR, endoscopic SAVR, robotic AVR, RAVR, patient-tailored surgical treatment

## Abstract

Surgical Aortic Valve Replacement (SAVR) is still one of the pillars of cardiac surgery practice, and its role is evolving into a more complex operation. The competition with structural valve therapies and the urgent demand for less invasive solutions have unleashed surgeons’ creativity in adapting to these new challenges. All the possible ways to surgically replace the aortic valve are analyzed in this review. Surgical techniques, advantages and disadvantages, and key differences are listed, helping surgeons navigate the available options. Sternotomy SAVR is the benchmark, but that is becoming obsolete and, in some cases, no longer performed for teaching purposes. Mini sternotomy is the easiest way to achieve minimal invasiveness in all anatomic situations, while right anterior thoracotomy is an elegant solution mastered by fewer surgeons. Endoscopic and robotic-assisted techniques are shaping the future of SAVR, yet they still lack wide adoption. The choice of approach is mainly dictated by the anatomic features of the patient and the surgeon’s skills. A flow diagram to overcome the learning curve and advance toward more complex surgery is provided here. Mastering as many techniques as possible is paramount when offering a patient-tailored approach and performing a safe and less invasive operation.

## 1. Introduction

While the adoption rate of transcatheter aortic valve replacement (TAVR) has outpaced that of surgical aortic valve replacement (SAVR), the volume of SAVR has continued to increase steadily over time, maintaining a risk-adjusted operative mortality rate of no more than 2% [1,2]. SAVR is still one of the pillars of cardiac surgery practice, and its role is evolving into a more complex operation. Two decades ago, a young surgeon would have expected to deal with a “straightforward” full sternotomy SAVR, yet today, their first isolated SAVR may be encountered as a TAVR explant performed in a minimally invasive fashion [3,4]. To overcome the pace of this terrific evolution, the key focus is patient-tailored surgical treatment [5]. Currently, several ways to accomplish SAVR have been demonstrated as safe and reproducible, but each approach has its advantages and disadvantages.

This review aims to analyze all the available surgical approaches to isolated SAVR, briefly summarizing the techniques and listing the pros and cons of each operation to guide surgeons in customizing the treatment strategy for each patient.

## 2. Methods

A comprehensive literature review was conducted to identify all surgical approaches to isolated SAVR, excluding transcatheter techniques. The search was performed using the PubMed, Scopus, Web of Science, and Cochrane Library databases, including results from their inception to 2024. The keywords included “aortic valve replacement”, “surgical aortic valve replacement”, “minimally invasive AVR”, “robotic AVR”, “full sternotomy AVR”, “Endoscopic AVR”, “Transcervical AVR”, and “right anterior thoracotomy AVR”.

The inclusion criteria were peer-reviewed studies, reviews, and case series describing surgical techniques, outcomes, and advancements in SAVR. Studies focused on isolated AVR, without concomitant procedures, were included. Non-English articles and studies involving TAVR were excluded.

Data extraction focused on surgical techniques (full sternotomy, partial sternotomy, right anterior thoracotomy, robotic-assisted approaches), patient outcomes, operative time, mortality, and long-term results. Narrative synthesis was performed to summarize the advantages, limitations, and clinical implications of each approach using the most pertinent and cited studies in the literature.

## 3. Sternotomy—The Benchmark for All Other “Cuts”

Although a long time has passed since the first modern SAVR was performed in Boston in 1960 by Dwight Harken [6], median sternotomy remains the gold standard approach, serving as the benchmark against which all other surgical techniques are evaluated [7,8]. The median sternotomy provides straightforward access for any type of cannulation, cardioplegia delivery, aortic root enlargement techniques, and the performance of any necessary concomitant surgical procedures. Despite its versatility, sternotomy SAVR is losing favor in the era of minimal invasiveness and TAVR, to the extent that residents are now being trained directly in minimally invasive techniques [9].

## 4. Mini Sternotomy—The Minimally Invasive Method That Fits All?

**Figure 1:**
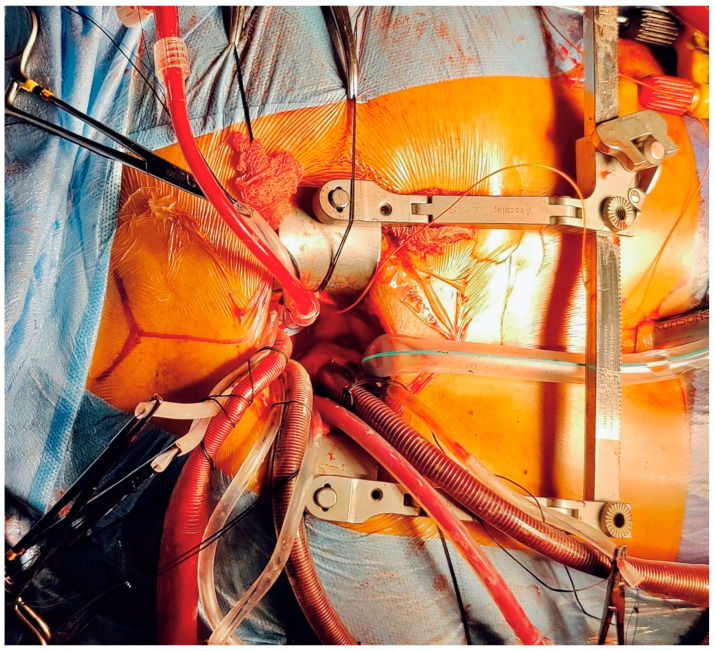
The smallest mini sternotomy (MS) SAVR achieved by the first author, with all central cannulation and a skin incision around 4 cm. (The arterial cannula is placed in the ascending aorta; the superior vena cava and right atrium are both used for optimal venous drainage; the de-airing root vent is in place; the left ventricular vent is in place via the right superior pulmonary vein; the subxiphoid drainage and pacing wires are already in place; the photo was taken while weaning off CPB).

Mini sternotomy (MS) was the first and more ancestral minimally invasive approach used for SAVR in the early 90s [10,11]. The intuitive concept on which MS relied was to spare the caudal half of the sternum, reducing surgical trauma and the instability of the rib cage created during full division of the sternum. Since its early adoption, MS has demonstrated high feasibility across a complete range of anatomic variations. MS ensures excellent exposure of the aortic root, enabling precise visualization and access for valve repair or replacement. Furthermore, MS provides safe and effective access to all essential surgical maneuvers typically performed during a full sternotomy [12]. Several arterial and venous cannulation strategies have been described, from full central to complete peripheral, and even bizarre configurations with only one superior vena cava cannula [13] (Figure 1). All types of solutions have been tried to partially split the sternum, such as “J”, “L”, “inverted L”, and “inverted T”. Currently, the most performed one is a “J” split on the right side at the third or fourth intercostal space with central arterial cannulation and right femoral percutaneous vein cannulation [14]. This latter approach closely resembles the full sternotomy that has also been used in aortic root repair or replacement, in aortic arch surgery, and even in the emergency setting of type A aortic dissection repair [15,16].

MS has been compared to conventional full sternotomy in several studies. Randomized controlled trials (RCTs) and meta-analyses have shown no significant difference in mortality between the two approaches [17,18]. MS is associated with slightly longer cross-clamp and bypass times, but shorter ICU and hospital stays [17,19]. Multiple studies have reported reduced blood loss and transfusion rates with MS [17,18]. While MS has been shown to be a safe alternative to full sternotomy during SAVR operations, the current evidence from RCTs is limited by small sample sizes and heterogeneity between studies [20]. Larger, well-designed randomized trials may be needed to definitively establish the role of MS in aortic valve surgery. However, a huge gap exists between the RCTs and real-world practice, in which the adoption of MS is so widespread that no one, neither the surgeon nor the patient, wants a complete split of the sternum during SAVR.

## 5. Right Anterior Thoracotomy

Although it appears to be a recently developed concept, SAVR by right anterior thoracotomy (RAT) was first described in a series of 50 patients in 1998. In this pioneering work, RAT was more of a sternal-sparing approach than a true minimally invasive approach. The skin incision was about 10 cm, with mobilization of two ribs and complete central cannulation [21]. The RAT technique has made a giant step forward since then, becoming an elegant and sophisticated sternal-sparing technique. The modern operative technique has its foundation in the “square-box principle” workspace [22]. A 5 cm skin incision is created at the third ICS, the right internal mammary artery is ligated, and the pericardium is opened under single lung ventilation. By placing several pericardial retraction sutures, the aorta is brought into proximity to the skin edges. The most crucial concept in this approach is the decision regarding which rib is dislocated, either the second or the third one. The decision is carried out by finger palpation of the aortic root position [23]. The operation is accomplished by complete femoral cannulation, either semi-Seldinger or percutaneous.

While some regard the RAT approach as feasible in all patients, others contend that its feasibility should be determined based on chest imaging. Van Praet et al. have meticulously described several patterns of ascending aorta–sternum position based on CT scans [24]. These patterns (Ia, Ib, II, and III) are labeled based on how much the ascending aorta is positioned leftward compared to the body of the sternum, with type III classified as not suitable for RAT. However, some authors affirm that 20 mmHg of continuous positive end-expiratory pressure in the left lung can correct even the most leftward dislocated aorta [22].

Multiple studies have concluded that RAT is a safe and effective minimally invasive alternative to median sternotomy. In a propensity-matched population undergoing sternotomy, RAT was linked to a reduced incidence of postoperative atrial fibrillation and blood transfusion, as well as shorter ventilation times and hospital stays [25]. RAT has improved patient satisfaction with cosmesis and has shown lower chest drain outputs compared to conventional sternotomy [26,27]. Glauber et al. reported favorable long-term outcomes, with a 94.8% survival rate observed at a follow-up of 31.5 months [28]. With the proper technique and experience, surgeons can safely perform RAT on a wide range of patients, offering a reproducible minimally invasive approach to aortic valve surgery [29].

## 6. Endoscopic SAVR

Although endoscopic SAVR (ESAVR) shares similarities with RAT SAVR, it should not be regarded as merely the same approach with the addition of a camera.

ESAVR was first described in 2014 by Vola et al., who used a five-port technique (two of them 20 mm in size) with a 3f Enable prosthesis. Two years later, the same group described a series using the Perceval S Sutureless valve [30,31].

Over the following years, several centers further developed and refined the ESAVR technique, reducing the size and number of ports used.

Tokoro et al. published the results from the first series of 47 patients on whom a three-port technique combined with a small 3 cm thoracotomy was performed without a rib spreader. This group was also the first to use a 3D scope, trying to “give back” perception of the depth to the surgeon [32]. After this group, Cresce et al., Yilmaz et al., and Hosoba et al. described larger series of ESAVR procedures with 125, 266, and 216 consecutive patients, respectively [33,34,35]. These series posed the basis for the modern ESAVR with a minimal port size (5 mm), minimally invasive extracorporeal circulation (MiECC), and peri-operative fast-track management. Overall, ESAVR has demonstrated low mortality rates, with 30-day mortality ranging from 0.5% to 2.0%. Conversion to sternotomy is rare, occurring in less than 1% of cases [34,35]. To facilitate the broader adoption of this approach, Danesi et al. meticulously outlined potential pitfalls and corresponding troubleshooting strategies to help surgeons master the ESAVR technique [36,37].

While the cosmetic benefits of endoscopic surgery over a small thoracotomy are relatively minor, the absence of a rib spreader has been reported to reduce the risk of post-thoracotomy pain syndrome [38]. Moreover, the endoscopic approach provides a broader field of view compared to direct vision through a small incision and is less impacted by anatomical variations among patients. In obese patients, adequate exposure of the target organ can still be achieved by advancing the scope deeper [32,34].

## 7. TransCervical SAVR—“A Transient Beacon of Innovation”

**Figure 2:**
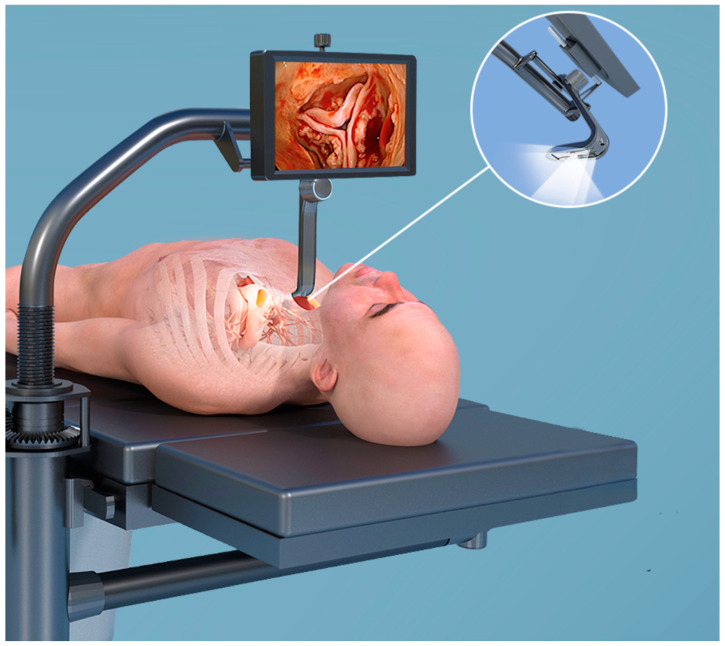
An animation of the CoreVista System (CardioPrecision Ltd., Glasgow, UK), a special retractor and illumination system used for the first-in-human transcervical SAVR.

The transcervical approach for thymectomy has demonstrated outstanding outcomes. This approach shows significantly reduced LOS and lower complication rates compared to traditional sternotomy techniques [39]. The enthusiasm for this novel approach has led to the development of the concept of transcervical SAVR. The rationale behind this new solution was to create truly minimal invasive access aiming to completely preserve the chest’s integrity while ensuring the effectiveness and benefits of a true surgical procedure for SAVR [40]. The pioneering team, led by Dapunt et al., developed this operative technique thanks to the “CoreVista System” (CardioPrecision Ltd., Glasgow, UK), a new surgical retractor designed specifically for this operation [40] (Figure 2).

Following extensive training with cadaver models, the first-in-human transcervical SAVR was successfully carried out in 2015. The operation was accomplished by using a 3f Enable sutureless valve [40]. The same team also demonstrated the feasibility of transcervical transaortic TAVR [41]. Although this innovative approach has been meticulously described [40] and was initially met with success at prestigious international congresses, no additional case series have been reported to date. The transcervical SAVR technique has not been adopted by other centers, potentially due to a lack of awareness among surgeons regarding the existence of this novel procedure.

## 8. Robotic AVR—Transitioning from Proof of Concept to a New Standard?

The concept of robotic-assisted SAVR (RAVR) is 20 years old. In the early 2000s, Folliguet et al. demonstrated the feasibility of this procedure by successfully performing five operations using a two-port technique combined with a 5 cm right thoracotomy [42,43]. The Mayo Clinic was the first to pioneer the integration of the Da Vinci (Intuitive, Sunnyvale, CA, USA) robotic system with the sutureless Perceval valve, aiming to further minimize the size of the thoracotomy incision [44]. In the early stages of RAVR, Nisivaco et al. took a significant step toward achieving a fully endoscopic operation by performing a series of five robotic-assisted closed-chest removals of aortic valve papillary fibroelastomas [45]. In 2020, Balkhy et al. reported the first fully endoscopic robotic-assisted aortic valve replacement using a sutureless valve. The procedure employed a four-port technique, consisting of two 8 mm ports, one 12 mm port, and one 25 mm port [46].

Badhwar et al. described the first consistent series of 20 RAVRs performed with some techniques adapted from their consolidated experience with robotic mitral valve surgery [47]. Their vision was to offer patients SAVR with a sutured prosthesis, either biological or mechanical, through the least invasive operation possible. They performed a 3 cm lateral thoracotomy at the fourth intercostal space, enabling the insertion of the prosthesis into the chest cavity. This approach preserved the pectoralis major, latissimus dorsi, and right internal thoracic artery while avoiding the rib damage associated with the RAT technique [47]. Additionally, through analysis of the intraoperative console times, the aortotomy closure was identified as the most difficult and time-consuming phase of the surgery. Badhwar et al. established a benchmark that other robotic programs could use as a foundation to initiate their own RAVR program, potentially mitigating the challenges of the initial steep learning curve [47].

In 2023 Yoshikawa et al. described the first Japanese experience with RAVR, and this year another landmark was achieved, namely fully endoscopic sutured RAVR [48,49].

Reinforcing the concept that the enhanced visualization of 3D cameras and degree of freedom offered by robotic consoles can be utilized to treat all patients, Badhwar et al. reignited the challenges of TAVR. A propensity-matched analysis of 144 pairs of low-risk patients undergoing either RAVR or TAVR showed the clear superiority of RAVR in terms of stroke, paravalvular leak, pacemaker implantation, and mortality [50].

**Figure 3:**
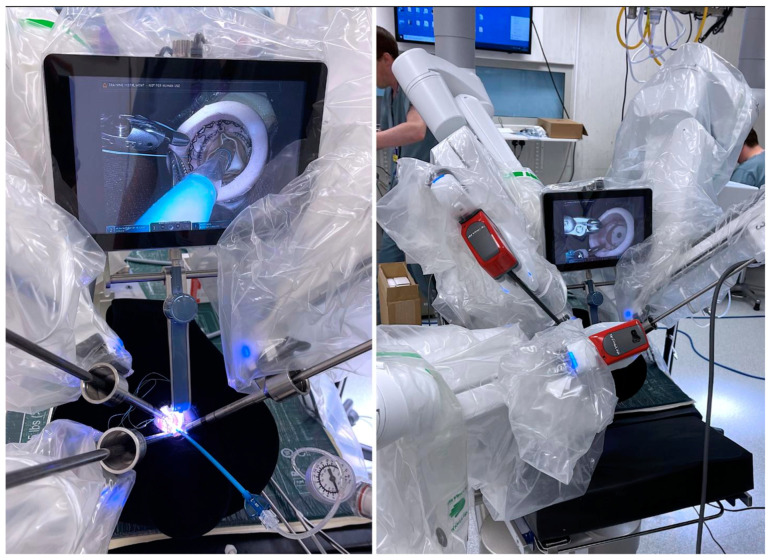
The proof of concept for a robot-assisted transcervical approach to aortic valve replacement by Sutherland et al. [51] (abstract presented at ISMICS 2023, Boston, MA, USA).

Pioneering new frontiers, Sutherland et al. presented a proof of concept for robotic-assisted uniportal transcervical SAVR, leveraging the CoreVista retractor system and a cross-specialty team to achieve a futuristic, totally endoscopic approach with enhanced visualization and dexterity, as well as minimally invasive neck access [51] (Figure 3). Could RAVR be emerging as the new standard of care?

**Figure 4:**
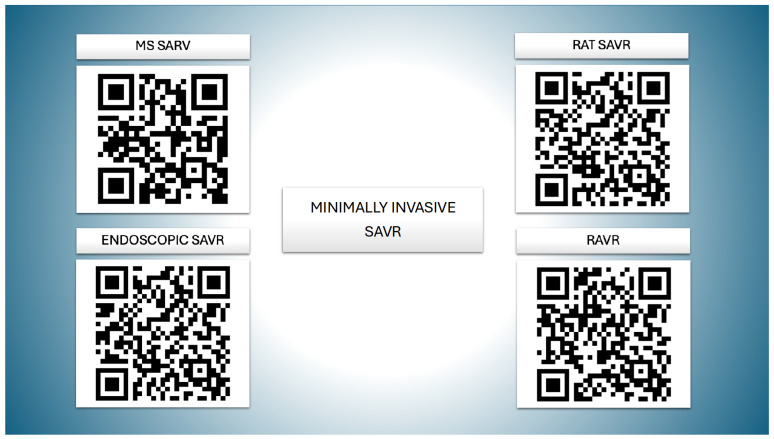
Each QR code is directly linked to some of the best surgical tutorials published in the Multimedia Manual of Cardio-Thoracic Surgery (EACTS)—MMCTS.org. SAVR—surgical aortic valve replacement; MS—mini sternotomy [52]; RAT—right anterior thoracotomy [53]; endoscopic SAVR [37]; RAVR—robotic-assisted aortic valve replacement [49].

Figure 4 offers readers a direct view of the surgical field in action. To enhance understanding, QR codes are provided, linking to some of the best peer-reviewed videos available. These resources give readers a quick and convenient opportunity to visualize and comprehend the procedures discussed in this review.

## 9. Sutureless Prostheses in MICS SAVR: “An Ace Up on Surgeon’s Sleeve”

Rapid deployment and sutureless prostheses have proven to be valuable and adaptable tools in complex surgical procedures [54]. Moreover, these prostheses have demonstrated excellent hemodynamic performance and long-term durability [55,56]. In minimally invasive surgery, these features make sutureless valves a strategic asset, assisting the surgeon in achieving two distinct goals.

First, sutureless prostheses aid the surgeon in navigating a restricted surgical field and situations where access to the aortic annulus for manual manipulation is challenging. It is no coincidence that many “firsts” in new minimally invasive approaches have been achieved using sutureless prostheses [30,31,40,46].

Since minimally invasive surgery requires surgeons to operate within a limited space, this has led to longer CPB and cross-clamp times, particularly during the learning curve. Sutureless valves have emerged as a promising option in this context. Studies have shown that sutureless valves significantly reduce aortic cross-clamp and cardiopulmonary bypass times compared to conventional bioprostheses [57,58]. The use of sutureless valves in minimally invasive SAVR has demonstrated excellent hemodynamic results, low mortality rates, and a reduced need for blood transfusions [58,59]. In particular, the use of sutureless valves can significantly reduce operative times in RAT SAVR [28,29]. Overall, sutureless valves appear to facilitate minimally invasive SAVR procedures, improving surgical outcomes and potentially expanding indications for less invasive approaches to aortic valve replacement.

## 10. Discussion—How to Choose the Better SAVR?

As previously discussed, SAVR can be accomplished safely through multiple operative techniques. The choice of the optimal approach remains a topic of ongoing debate, and a definitive solution appears to be far from being reached.

The first question to be addressed when comparing two different surgical strategies is safety. However, neither of the aforementioned approaches have demonstrated clear superiority when compared to the benchmark outcomes of sternotomy. In a large network meta-analysis involving 15,000 patients comparing MS, RAT, and sternotomy SAVR, MS demonstrated a slight advantage in terms of short-term mortality; however, no significant differences were observed between the long-term outcomes [60]. Even when analyzed through a Bayesian network meta-analysis, no significant safety differences have been identified between MS and RAT SAVR [61]. It is important to note that this statistical method has faced significant criticism, as even small differences among studies can lead to biased results [62].

Therefore, the focus should move on to other metrics of comparison, such as anatomical suitability, CPB and cross-clamp time (XC), blood loss and transfusions, respiratory complications, post-operative pain, and even cosmetic results.

All minimally invasive approaches demonstrated an increase in CPB and XC times compared to sternotomy due to their inherently higher technical demands. On the other hand, all minimally invasive SAVRs showed lower blood loss and shorter lengths of stay [63]. ESAVR and RAVR are such advanced techniques that the comparison has been done only with MS or RAT. ESAVR exhibited longer CPB and XC times compared to MS; however, perioperative bleeding was lower in ESAVR, and no other significant differences were observed [64]. When it comes to respiratory complications, Bacchi et al. report that maintaining pleural integrity positively impacts the short- and long-term outcomes of patients undergoing minimally invasive SAVR [65]. Post-operative pain is significantly reduced simply by avoiding rib spreading, underscoring the importance of the effort to strive for ESAVR and RAVR [38,66].

It seems that in the right hands, each type of SAVR is feasible and safe. Is it perhaps more a matter of the craftsman’s skill rather than the operative technique itself [67]?

**Figure 5:**
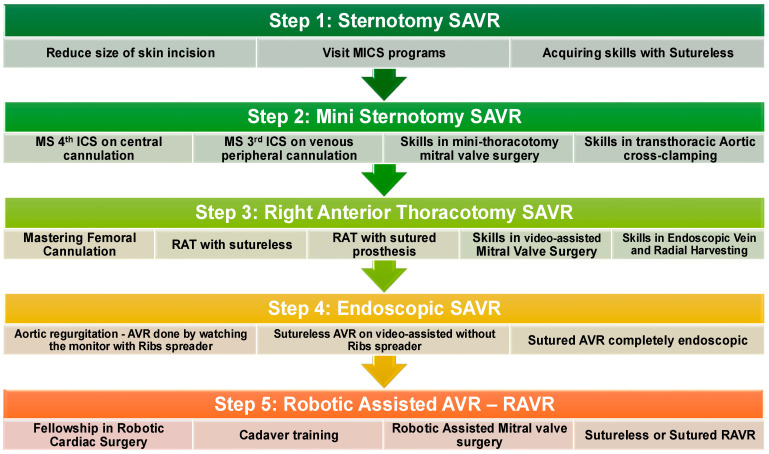
A flow diagram of an ideal pathway to transition from standard full sternotomy SAVR to RAVR. Each step highlights the skills and training required to move toward the next step safely. SAVR—surgical aortic valve replacement; MICS—minimally invasive cardiac surgery; MS—mini sternotomy; ICS—intercostal space; RAT—right anterior thoracotomy; AVR—aortic valve replacement; RAVR—robotic-assisted aortic valve replacement.

Each of the previously mentioned large series for a given technique was published only after years of meticulous refinement and skill development. The pathway to mastering these techniques is long and should begin as early as possible in the training process [34]. Transitioning from simpler to more technically demanding types of SAVR requires significant dedication and patience, which are needed to successfully overcome the learning curve. A flow diagram proposing an ideal progression is shown in Figure 5. Some of the most important steps in achieving proficiency in minimally invasive SARV are mastering femoral cannulation, gaining expertise with sutureless prostheses, and acquiring a profound knowledge of surgical instruments (retractors, long-shafted customized tools, etc.). Extensive cadaveric lab and simulation training appear to be essential for developing endoscopic skills.

How can a surgeon choose what is the better approach? By tailoring the SAVR approach to the patient’s anatomy, comorbidities, and even cosmetic preference. Perhaps a patient with COPD and peripheral vascular disease will benefit from MS, which spares pleura and femoral vessels. An obese patient candidate for RAT will benefit more from ESAVR or RAVR due to the easier visualization of the chest anatomy. Moreover, a patient with severe aortic regurgitation and a reduced ejection fraction might benefit more from the faster sternotomy approach, as it minimizes CPB and XC times as much as possible.

In the era of TAVR and minimally invasive procedures, surgeons should move away from a “one-size-fits-all” approach to SAVR and instead adopt a personalized surgical strategy. Achieving this requires mastering a wide range of operative techniques.

## 11. Conclusions

SAVR can be accomplished through several operative strategies that require different levels of skill and surgical experience. Currently, none of the existing approaches have demonstrated clear superiority, yet each kind of operation has its advantages and disadvantages. Only some operative strategies show feasibility in all comers, independent of the anatomy of the patient. The transition from standard open sternotomy to minimal invasiveness and then full endoscopic approaches should follow key steps such as mastering femoral cannulation and developing skills in video-assisted surgery. A paramount role is also played by sutureless prostheses in selected cases. The surgeon of the modern era should ideally master at least two or three different approaches in order to be capable of offering patients the true advantages of SAVR through the least invasive operation tailored to the patient’s anatomy and comorbidities.

## Data Availability

These data were derived from the following resources available in the public domain: PubMed/Medline; EMBASE; Google Scholar.

## References

[1] Bowdish M.E., D’Agostino R.S., Thourani V.H., Desai N., Shahian D.M., Fernandez F.G., Badhwar V. (2020). The Society of Thoracic Surgeons Adult Cardiac Surgery Database: 2020 Update on Outcomes and Research. Ann. Thorac. Surg..

[2] Cabrucci F., Baudo M., Yamashita Y., Dokollari A., Sicouri S., Ramlawi B. (2024). Short and Long-Term Outcomes of Transcatheter Aortic Valve Implantation in the Small Aortic Annulus: A Systematic Literature Review. J. Pers. Med..

[3] Sharma T., Krishnan A.M., Lahoud R., Polomsky M., Dauerman H.L. (2022). National Trends in TAVR and SAVR for Patients With Severe Isolated Aortic Stenosis. J. Am. Coll. Cardiol..

[4] Bowdish M.E., Habib R.H., Kaneko T., Thourani V.H., Badhwar V. (2024). Cardiac Surgery After Transcatheter Aortic Valve Replacement: Trends and Outcomes. Ann. Thorac. Surg..

[5] Bonacchi M., Bacchi B., Cabrucci F., Tevaearai Stahel H., Jeenchen Chen R., Dokollari A. (2023). Editorial: Insights in heart surgery: 2022. Front. Cardiovasc. Med..

[6] Kuijpers P.M.J.C. History in Medicine: The Aortic Valve. https://www.escardio.org/Journals/E-Journal-of-Cardiology-Practice/Volume-18/history-in-medicine-the-aortic-valve.

[7] Bowdish M.E., Hui D.S., Cleveland J.D., Mack W.J., Sinha R., Ranjan R., Cohen R.G., Baker C.J., Cunningham M.J., Barr M.L. (2016). A comparison of aortic valve replacement via an anterior right minithoracotomy with standard sternotomy: A propensity score analysis of 492 patients. Eur. J. Cardio-Thorac. Surg. Off. J. Eur. Assoc. Cardio-Thorac. Surg..

[8] Furukawa N., Kuss O., Aboud A., Schönbrodt M., Renner A., Hakim Meibodi K., Becker T., Zittermann A., Gummert J.F., Börgermann J. (2014). Ministernotomy versus conventional sternotomy for aortic valve replacement: Matched propensity score analysis of 808 patients. Eur. J. Cardio-Thorac. Surg. Off. J. Eur. Assoc. Cardio-Thorac. Surg..

[9] Gerfer S., Eghbalzadeh K., Brinkschröder S., Djordjevic I., Rustenbach C., Rahmanian P., Mader N., Kuhn E., Wahlers T. (2023). Is It Reasonable to Perform Isolated SAVR by Residents in the TAVI Era?. Thorac. Cardiovasc. Surg..

[10] Izzat M.B., Yim A.P., El-Zufari M.H., Khaw K.S. (1998). Upper T mini-sternotomy for aortic valve operations. Chest.

[11] Szerafin T., Jagamos E., Jaber O., Horváth A., Horváth G., Olin C., Péterffy A. (1997). Mini-sternotomy for aortic valve surgery. Acta Chir. Hung..

[12] Gundry S.R., Shattuck O.H., Razzouk A.J., del Rio M.J., Sardari F.F., Bailey L.L. (1998). Facile minimally invasive cardiac surgery via ministernotomy. Ann. Thorac. Surg..

[13] Pfeiffer S., Fischlein T., Vogt F., Santarpino G. (2015). Superior vena cava cannulation in aortic valve surgery: An alternative strategy for a hemisternotomy approach. Interact. Cardiovasc. Thorac. Surg..

[14] Van Praet K.M., Nersesian G., Kukucka M., Kofler M., Wert L., Klein C., Unbehaun A., Kempfert J., Falk V. (2022). Minimally invasive surgical aortic valve replacement via a partial upper ministernotomy. Multimed. Man. Cardiothorac. Surg. MMCTS.

[15] Bonacchi M., Cabrucci F., Bacchi B., Haranal M., Gelsomino S., Ramlawi B., Dokollari A. (2022). Editorial: Novel insights into aortic arch repair. Front. Cardiovasc. Med..

[16] Cabrucci F., Pellegrini G., Bacchi B., Ferrara F., Balestracci P., Petrone D., Bessi G., Codecasa R. (2024). Minimally invasive type A aortic dissection repair: Aortic valve resuspension with neomedia creation and the Ascyrus Medical Dissection Stent. Multimed. Man. Cardiothorac. Surg. MMCTS.

[17] Brown M.L., McKellar S.H., Sundt T.M., Schaff H.V. (2009). Ministernotomy versus conventional sternotomy for aortic valve replacement: A systematic review and meta-analysis. J. Thorac. Cardiovasc. Surg..

[18] Harky A., Al-Adhami A., Chan J.S.K., Wong C.H.M., Bashir M. (2019). Minimally Invasive Versus Conventional Aortic Root Replacement—A Systematic Review and Meta-Analysis. Heart Lung Circ..

[19] Khoshbin E., Prayaga S., Kinsella J., Sutherland F.W.H. (2011). Mini-sternotomy for aortic valve replacement reduces the length of stay in the cardiac intensive care unit: Meta-analysis of randomised controlled trials. BMJ Open.

[20] Kirmani B.H., Jones S.G., Malaisrie S.C., Chung D.A., Williams R.J. (2017). Limited versus full sternotomy for aortic valve replacement. Cochrane Database Syst. Rev..

[21] Minale C., Reifschneider H.J., Schmitz E., Uckmann F.P. (1998). Minimally invasive aortic valve replacement without sternotomy. Experience with the first 50 cases. Eur. J. Cardio-Thorac. Surg. Off. J. Eur. Assoc. Cardio-Thorac. Surg..

[22] Ribeiro I.B., Ruel M. (2019). Right Anterior Minithoracotomy for Aortic Valve Replacement: A Widely Applicable, Simple, and Stepwise Approach. Innovations.

[23] Issa H.M.N., Ruel M. (2024). Minimally invasive aortic valve replacement through a right anterior thoracotomy. Multimed. Man. Cardiothorac. Surg. MMCTS.

[24] Van Praet K.M., van Kampen A., Kofler M., Richter G., Sündermann S.H., Meyer A., Unbehaun A., Kurz S., Jacobs S., Falk V. (2020). Minimally invasive surgical aortic valve replacement: The RALT approach. J. Card. Surg..

[25] Glauber M., Miceli A., Gilmanov D., Ferrarini M., Bevilacqua S., Farneti P.A., Solinas M. (2013). Right anterior minithoracotomy versus conventional aortic valve replacement: A propensity score matched study. J. Thorac. Cardiovasc. Surg..

[26] Ahangar A.G., Charag A.H., Wani M.L., Ganie F.A., Singh S., Ahmad Qadri S.A., Ahmad Shah Z. (2013). Comparing Aortic Valve Replacement through Right Anterolateral Thoracotomy with Median Sternotomy. Int. Cardiovasc. Res. J..

[27] Seitz M., Goldblatt J., Paul E., Marcus T., Larobina M., Yap C.-H. (2019). Minimally Invasive Aortic Valve Replacement Via Right Anterior Mini-Thoracotomy: Propensity Matched Initial Experience. Heart Lung Circ..

[28] Glauber M., Gilmanov D., Farneti P.A., Kallushi E., Miceli A., Chiaramonti F., Murzi M., Solinas M. (2015). Right anterior minithoracotomy for aortic valve replacement: 10-year experience of a single center. J. Thorac. Cardiovasc. Surg..

[29] Bouchot O., Petrosyan A., Morgant M.C., Malapert G. (2018). Technical points for aortic valve replacement through right anterior minithoracotomy. Eur. J. Cardio-Thorac. Surg. Off. J. Eur. Assoc. Cardio-Thorac. Surg..

[30] Vola M., Fuzellier J.-F., Chavent B., Duprey A. (2014). First human totally endoscopic aortic valve replacement: An early report. J. Thorac. Cardiovasc. Surg..

[31] Vola M., Fuzellier J.-F., Gerbay A., Campisi S. (2016). First in Human Totally Endoscopic Perceval Valve Implantation. Ann. Thorac. Surg..

[32] Tokoro M., Sawaki S., Ozeki T., Orii M., Usui A., Ito T. (2020). Totally endoscopic aortic valve replacement via an anterolateral approach using a standard prosthesis. Interact. Cardiovasc. Thorac. Surg..

[33] Cresce G.D., Sella M., Hinna Danesi T., Favaro A., Salvador L. (2020). Minimally Invasive Endoscopic Aortic Valve Replacement: Operative Results. Semin. Thorac. Cardiovasc. Surg..

[34] Yilmaz A., Van Genechten S., Claessens J., Packlé L., Maessen J., Kaya A. (2022). A totally endoscopic approach for aortic valve surgery. Eur. J. Cardio-Thorac. Surg. Off. J. Eur. Assoc. Cardio-Thorac. Surg..

[35] Hosoba S., Ito T., Mori M., Kato R., Kajiyama K., Maeda S., Nakai Y., Morishita Y. (2023). Endoscopic Aortic Valve Replacement: Initial Outcomes of Isolated and Concomitant Surgery. Ann. Thorac. Surg..

[36] Hinna Danesi T., Salvador L. (2018). Minimally invasive aortic valve replacement techniques using endoscopic surgery: “must dos” and “preferences”. Eur. J. Cardio-Thorac. Surg. Off. J. Eur. Assoc. Cardio-Thorac. Surg..

[37] Danesi T.H. (2023). Different valve types: Tips and tricks for a totally endoscopic aortic valve replacement. Multimed. Man. Cardiothorac. Surg. MMCTS.

[38] Yamashita Y., Mukaida H., Harada H., Tsubokawa N. (2013). Post-thoracotomy pain and long-term survival associated with video-assisted thoracic surgery lobectomy methods for clinical T1N0 lung cancer: A patient-oriented, prospective cohort study. Eur. J. Cardio-Thorac. Surg. Off. J. Eur. Assoc. Cardio-Thorac. Surg..

[39] Shrager J.B. (2010). Extended transcervical thymectomy: The ultimate minimally invasive approach. Ann. Thorac. Surg..

[40] Dapunt O.E., Luha O., Ebner A., Sonecki P., Spadaccio C., Sutherland F.W.H. (2016). First-in-Man Transcervical Surgical Aortic Valve Replacement Using the CoreVista System. Innovations.

[41] Dapunt O.E., Luha O., Ebner A., Sonecki P., Spadaccio C., Sutherland F.W.H. (2016). New Less Invasive Approach for Direct Aortic Transcatheter Aortic Valve Replacement Using Novel CoreVista Transcervical Access System. JACC Cardiovasc. Interv..

[42] Folliguet T.A., Vanhuyse F., Magnano D., Laborde F. (2004). Robotic aortic valve replacement: Case report. Heart Surg. Forum.

[43] Folliguet T.A., Vanhuyse F., Konstantinos Z., Laborde F. (2005). Early experience with robotic aortic valve replacement. Eur. J. Cardio-Thorac. Surg. Off. J. Eur. Assoc. Cardio-Thorac. Surg..

[44] Suri R.M., Burkhart H.M., Schaff H.V. (2010). Robot-assisted aortic valve replacement using a novel sutureless bovine pericardial prosthesis: Proof of concept as an alternative to percutaneous implantation. Innovations.

[45] Nisivaco S.M., Patel B., Balkhy H.H. (2019). Robotic totally endoscopic excision of aortic valve papillary fibroelastoma: The least invasive approach. J. Card. Surg..

[46] Balkhy H.H., Kitahara H. (2020). First Human Totally Endoscopic Robotic-Assisted Sutureless Aortic Valve Replacement. Ann. Thorac. Surg..

[47] Badhwar V., Wei L.M., Cook C.C., Hayanga J.W.A., Daggubati R., Sengupta P.P., Rankin J.S. (2021). Robotic aortic valve replacement. J. Thorac. Cardiovasc. Surg..

[48] Yoshikawa Y., Kishimoto Y., Onohara T., Kumagai K., Nii R., Sumi N., Kishimoto N., Ikeda Y., Yoshikawa Y., Yamane K. (2023). Robot-Assisted Aortic Valve Replacement—First Clinical Report in Japan. Circ. J. Off. J. Jpn. Circ. Soc..

[49] Arai A., Kitahara H., Balkhy H.H. (2024). Robotic totally endoscopic aortic valve replacement with a sutured bioprosthesis. Multimed. Man. Cardiothorac. Surg. MMCTS.

[50] Jagadeesan V., Mehaffey J.H., Darehzereshki A., Alharbi A., Kawsara M., Daggubati R., Wei L., Badhwar V. (2024). Robotic Aortic Valve Replacement versus Transcatheter Aortic Valve Replacement: A Propensity Matched Analysis. Ann. Thorac. Surg..

[51] International Society for Minimally Invasive Cardiothoracic Surgery. https://ismics.org.

[52] Reser D., Holubec T., Scherman J., Yilmaz M., Guidotti A., Maisano F. (2015). Upper ministernotomy. Multimed. Man. Cardiothorac. Surg. MMCTS.

[53] Van Praet K.M., Van Kampen A., Kofler M., Unbehaun A., Hommel M., Jacobs S., Falk V., Kempfert J. (2020). Minimally invasive surgical aortic valve replacement through a right anterolateral thoracotomy. Multimed. Man. Cardiothorac. Surg. MMCTS.

[54] Cabrucci F., Bacchi B., Codecasa R., Stefàno P. (2023). Case report: Infective endocarditis after transcatheter aortic valve implantation surgically treated with sutureless prosthesis and ascending aorta replacement. Front. Cardiovasc. Med..

[55] Dokollari A., Torregrossa G., Sicouri S., Veshti A., Margaryan R., Cameli M., Mandoli G.E., Maccherini M., Montesi G., Cabrucci F. (2022). Pearls, pitfalls, and surgical indications of the Intuity TM heart valve: A rapid deployment bioprosthesis. A systematic review of the literature. J. Card. Surg..

[56] Dokollari A., Torregrossa G., Bisleri G., Hassanabad A.F., Sa M.P., Sicouri S., Veshti A., Prifti E., Bacchi B., Cabrucci F. (2023). Early and Long-Term Clinical and Echocardiographic Outcomes of Sutureless vs. Sutured Bioprosthesis for Aortic Valve Replacement. J. Cardiovasc. Dev. Dis..

[57] Vola M., Campisi S., Gerbay A., Fuzellier J.-F., Ayari I., Favre J.-P., Faure M., Morel J., Anselmi A. (2015). Sutureless prostheses and less invasive aortic valve replacement: Just an issue of clamping time?. Ann. Thorac. Surg..

[58] Paparella D., Santarpino G., Moscarelli M., Guida P., De Santis A., Fattouch K., Martinelli L., Coppola R., Mikus E., Albertini A. (2021). Minimally invasive aortic valve replacement: Short-term efficacy of sutureless compared with stented bioprostheses. Interact. Cardiovasc. Thorac. Surg..

[59] Miceli A., Santarpino G., Pfeiffer S., Murzi M., Gilmanov D., Concistré G., Quaini E., Solinas M., Fischlein T., Glauber M. (2014). Minimally invasive aortic valve replacement with Perceval S sutureless valve: Early outcomes and one-year survival from two European centers. J. Thorac. Cardiovasc. Surg..

[60] Ogami T., Yokoyama Y., Takagi H., Serna-Gallegos D., Ferdinand F.D., Sultan I., Kuno T. (2022). Minimally invasive versus conventional aortic valve replacement: The network meta-analysis. J. Card. Surg..

[61] Phan K., Xie A., Tsai Y.-C., Black D., Di Eusanio M., Yan T.D. (2015). Ministernotomy or minithoracotomy for minimally invasive aortic valve replacement: A Bayesian network meta-analysis. Ann. Cardiothorac. Surg..

[62] Esterhuizen T.M., Thabane L. (2016). Con: Meta-analysis: Some key limitations and potential solutions. Nephrol. Dial. Transplant. Off. Publ. Eur. Dial. Transpl. Assoc.—Eur. Ren. Assoc..

[63] Almeida A.S., Ceron R.O., Anschau F., de Oliveira J.B., Leão Neto T.C., Rode J., Rey R.A.W., Lira K.B., Delvaux R.S., de Souza R.O.R.R. (2022). Conventional Versus Minimally Invasive Aortic Valve Replacement Surgery: A Systematic Review, Meta-Analysis, and Meta-Regression. Innovations.

[64] Yilmaz A., Claessens J., Packlé L., Van Genechten S., Dönmez K., Awouters C., Herbots L. (2023). Aortic Valve Replacement: Totally Endoscopic versus Mini-Sternotomy. J. Clin. Med..

[65] Bacchi B., Cabrucci F., Chiarello B., Dokollari A., Bonacchi M. (2024). Impact of Pleural Integrity Preservation After Minimally Invasive Aortic Valve Surgery. Innovations.

[66] Dokollari A., Sicouri S., Erten O., Ramlawi B., Cameli M., Mandoli G.E., Bacchi B., Cabrucci F., Sutter F.P., Kjelstrom S. (2023). Clinical Outcomes of Cryo Nerve Ablation Technique for Pain Management: An Exploratory Study in Patients Undergoing Left Thoracotomy Coronary Artery Bypass Grafting. Rev. Cardiovasc. Med..

[67] Bonacchi M., Cabrucci F., Bacchi B., Dokollari A. (2023). Reply from authors: Dazzling like an art(ist), worthwhile like a craftsman. JTCVS Open.

